# Calreticulin as an Adjuvant *In Vivo* to Promote Dendritic Cell Maturation and Enhance Antigen-Specific T Lymphocyte Responses against Melanoma

**DOI:** 10.1155/2022/8802004

**Published:** 2022-08-09

**Authors:** Zheng Gong, Ming Chen, Jie Miao, Chao-Jie Han, Qiao Zhong, Fang-Yuan Gong, Xiao-Ming Gao

**Affiliations:** ^1^Institute of Biology and Medical Sciences, School of Biology and Basic Medical Sciences, Soochow University, Suzhou 215123, China; ^2^Department of Laboratory Medicine, The Affiliated Suzhou Hospital of Nanjing Medical University, Gusu School, Nanjing Medical University, Suzhou 215002, China; ^3^Key Laboratory of Systemic Biomedical Study, Suzhou 215123, China

## Abstract

An endoplasmic reticulum resident protein, calreticulin (CRT), participates in many cellular processes. CRT is a tumor-associated antigen with an important role in antitumor immunity. Previously, we reported that the recombinant CRT fragment 39-272 (CRT/39-272) exhibited superior immunobiological activity, activating macrophages to release cytokines and promoting dendritic cell (DC) maturation. However, the effect of CRT/39-272 *in vivo*, especially its adjuvant effect on *in vivo* antitumor immune responses, was not fully investigated. In this study, we constructed a fusion protein linking CRT/39-272 to an ovalbumin (OVA) peptide (residues 182–297, OVAp) and used the fusion protein (OVAp-CRT) to examine the adjuvant effect of CRT. We investigated whether CRT/39-272 could induce bone marrow-derived DC maturation and strongly promote the proliferation of OVA-specific T cells *in vitro*. Compared with OVAp, OVAp-CRT induced stronger antigen-specific T lymphocyte responses, including antigen-specific T cell proliferation, interferon-*γ* secretion, and cytotoxic T lymphocyte responses. OVAp-CRT-immunized mice generated significantly increased OVAp-specific antibody and CD4^+^/CD8^+^ memory T cells, which mediated long-term protective effects. OVAp-CRT upregulated CD40, CD80, and CD86 expressions in splenic conventional DCs. Furthermore, OVAp-CRT protected immunized mice against OVA-expressing B16 melanoma cells *in vivo*. Moreover, mice that were adoptively transferred with OVAp-CRT-pulsed DCs showed inhibited tumor growth and prolonged mouse survival. Our results demonstrate that CRT/39-272 can be used as a potential new adjuvant for tumor vaccines, and this finding may be useful in tumor vaccine development.

## 1. Introduction

Calreticulin (CRT) is a 46 kDa endoplasmic reticulum resident protein that contains three domains: a lectin-like N domain (residues 1–197), a proline-rich P domain (residues 198–308), and a calcium-binding C domain (residues 309–416) [1–5]. CRT participates in multiple cellular processes, including maintaining intracellular Ca^2+^ homeostasis, assisting proper protein folding, and participating in major histocompatibility complex (MHC) class I assembly [5–7]. In addition, an increasing number of studies have demonstrated that CRT is a tumor-associated antigen. CRT is significantly upregulated in various tumor cells (e.g., melanoma) and soluble CRT (sCRT) is present in the body fluids of patients with different cancers [8–10]. CRT is recognized as a heat shock protein (HSP), exhibiting potent immunobiological activity [1]. Previously, we demonstrated that a recombinant fragment of murine CRT (rCRT/39-272) covering its partial N and P domains is a potent activator of B cells and macrophages [11]. CRT/39-272 also induced dendritic cell (DC) activation and maturation *via* the Toll-like receptor 4 (TLR4) and CD14 pathways [12]. When fused to enhanced green fluorescence protein (EGFP), CRT/39-272 greatly improved humoral responses against EGFP in both BALB/c and T cell-deficient nude mice [11]. Our previous findings also indicated that CRT enhances immunoglobulin G (IgG) responses to the SARS-CoV (severe acute respiratory syndrome coronavirus) spike protein [13]. Investigations involving DNA vaccines encoding CRT and target antigen fusion proteins have revealed that CRT can function as a molecular adjuvant [14–16].

DCs are the most optimal antigen-presenting cells (APCs) and can elicit primary T cell responses [17–19]. DCs bridge innate immunity and adaptive immunity. Based on their potent antigen presentation and T cell activation activity, DCs have subsequently been tested as cancer vaccines, where they have been loaded with tumor antigens and used to promote tumor antigen-specific antitumor T cell immunity [20]. DCs can also produce many cytokines and chemokines to enhance antitumor responses. Some adjuvants have been used in combination to improve the therapeutic efficacy of DC-based vaccines [21, 22]. Among these adjuvants, more attention has been focused on HSPs that can potently stimulate the cytotoxic T lymphocyte (CTL) and Th1-polarized response [23–25]. CRT can act as an adjuvant to promote DC maturation and enhance antigen-specific CTL responses against non-small-cell lung cancer cells *in vitro* [26]. Nevertheless, its adjuvant effect on *in vivo* antitumor immune responses is not fully understood.

In this study, we used ovalbumin- (OVA-) expressing B16 melanoma cells (B16-OVA) as a tumor model, where OVA can be recognized as a tumor-specific antigen. We fused CRT/39-272 with OVA/182-297 (OVAp), an OVA fragment containing SIINFEKL (an H2-K^b^ restricted CTL epitope), and generated the OVAp-CRT recombinant fusion protein. We investigated whether CRT could enhance antigen presentation and the fusion protein could activate DCs and whether an OVA-specific antitumor response could be induced *in vivo* against B16-OVA cells. We also evaluated the antitumor effect derived from DCs pulsed with the fusion protein. CRT/39-272 promoted a stronger antitumor response than OVAp alone *in vivo*. These results demonstrate that CRT/39-272 can be a potential new adjuvant for tumor vaccines.

## 2. Materials and Methods

### 2.1. Cell Lines

The B16 melanoma cell line was purchased from the Cell Bank of the Chinese Academy of Sciences (Shanghai, China). The B16-OVA cells were generously provided by Dr. H. Liu (Soochow University, Suzhou, China). The cells were cultured in Dulbecco's modified Eagle's medium supplemented with 10% fetal bovine serum and 100 U/mL penicillin/streptomycin (Invitrogen, USA). All cell lines were cultured at 37°C in a humidified atmosphere of 5% CO_2_.

### 2.2. Mice

Female C57BL/6 (H-2^b^) mice (6–8 weeks old) were purchased from the Model Animal Research Center of Nanjing University (Nanjing, China). OT-I mice (transgenic for T cell receptors against chicken OVA peptide 257–264) were generously provided by Dr. H. Liu (Soochow University, Suzhou, China). The mouse experimental procedures were performed under specific pathogen-free conditions. All experimental procedures were approved by the local ethical review process committee of Soochow University.

### 2.3. Recombinant Proteins

Preparation of the expression vector pET28a-CRT/39-272 encoding murine CRT/39-272 has been described previously [11]. For constructing the vector pET28a-OVAp, total cellular RNA was extracted from B16-OVA cells. RNA was isolated from the cells with a Total RNA Kit II (Omega Bio-Tek, USA) and reverse-transcribed into complementary DNA (cDNA) using an oligo (dT) primer (Takara, Japan). The resultant cDNA was used as a template for PCR amplification of the gene encoding protein OVA fragment 182–297 using forward primer 5′-TAGGATCCGTCCTTCAGCCAAGCTCCGTGG-3′ and reverse primer 5′-CGAAGCTTCTCTTCCATAACATTAGA-3′. The sequencing-verified DNA product was inserted into a pET28a expression vector, and the recombinant protein expressed contained a 15-amino acid histidine (His6) tag. Overlapping extension PCR was performed to prepare DNA encoding the OVAp and CRT/39-272 fusion protein, i.e., OVAp-CRT, with the linker sequence Gly-Pro-Gly. The vector pET28a-CRT/39-272 was used as a template for PCR amplification of CRT/39-272 using forward primer 5′-CGGGATCCGAATCCAAACATAAGTCCG-3′ and reverse primer 5′-GCTTGGCTGAAGGACTCCAGGTCCCTTGTATTCAGGATT-3′. The vector pET28a-OVAp was used as a template for PCR amplification of OVAp using forward primer 5′-AATCCTGAATACAAGGGACCTGGAGTCCTTCAGCCAAGC-3′ and reverse primer 5′-CGAAGCTTCTCTTCCATAACATTAGA-3′. The fusion gene was PCR-amplified using the two earlier DNA products as the template and with forward primer 5′-CGGGATCCGAATCCAAACATAAGTCCG-3′ and reverse primer 5′-CGAAGCTTCTCTTCCATAACATTAGA-3′. The amplified product was then cloned into the *Bam*HI/*Hin*dIII sites of pET28a-OVAp-CRT vectors. The His6-tagged recombinant proteins were expressed in *Escherichia coli* BL21 (DE3) (CWBio, China) and purified using nickel- (Ni-) nitrilotriacetic acid resin (GE Healthcare, USA) following the manufacturers' instructions. All proteins were desalted using snakeskin dialysis tubing (Thermo Fisher Scientific, USA) in phosphate-buffered saline (PBS). The protein concentration was determined using a bicinchoninic acid protein assay reagent (Pierce, USA). All recombinant proteins used in this study were >90% purity as evaluated by Coomassie blue-stained sodium dodecyl sulfate- (SDS-) polyacrylamide gels.

### 2.4. Western Blotting

The protein bands separated in SDS-PAGE (polyacrylamide gel electrophoresis) were electroblotted onto polyvinylidene fluoride (PVDF) membranes at a constant current of 350 mA in trans buffer (50 mM Tris (pH 8.0) containing 0.192 M glycine and 20% methanol), using a Bio-Rad Trans-Blot Cell (Bio-Rad, USA). The membranes were incubated for 1 h at room temperature in PBST (PBS containing 0.05% Tween 20) containing 5% nonfat dry milk, followed by overnight incubation with the mouse anti-CRT monoclonal antibodies (mAb153.24, provided by Dr. B. Jin, Fourth Military Medical University, Xi'an, China) or rabbit anti-OVA polyclonal antibodies (Bioss Antibodies, USA) at 4°C with constant agitation. After three times washes with PBST, the membranes were incubated for 1 h with Alexa Fluor 680-conjugated secondary antibody (Jackson ImmunoResearch, USA) and visualized using the Odyssey infrared imaging system (LI-COR Biosciences, USA).

### 2.5. Flow Cytometric Analysis

Freshly isolated splenocytes and cells from blood or inguinal lymph nodes (ILN) were stained with APC-conjugated rat anti-mouse CD4, FITC-conjugated rat anti-mouse CD8a, PE-conjugated rat anti-mouse CD44, APC-conjugated rat anti-mouse CD62L, and APC-conjugated Armenian hamster anti-mouse CD11c, or APC-, PE-, and FITC-conjugated isotype control Abs (all from BioLegend, USA) for 30 min at 4°C. After washing, the cell surface markers were determined by using Attune NxT flow cytometer (Life Technology, USA).

### 2.6. Enzyme-Linked Immunosorbent Assay (ELISA) of Interleukin- (IL-) 12/IL-23 (p40)

The cell culture supernatants were collected and centrifuged at 12,000 × *g* for 1 min to remove cellular debris. The concentration of mouse IL-12/IL-23 (p40) was evaluated by using the commercial ELISA kit (Invitrogen, USA) according to the manufacturer's protocols.

### 2.7. Cell Preparation and Culture of Bone Marrow-Derived DCs (BMDCs)

Bone marrow was flushed from the femurs and tibiae of the mice and incubated at a starting concentration of 1 × 10^6^ cells/mL in RPMI 1640 medium containing 20 ng/mL recombinant mouse granulocyte monocyte–colony-stimulating factor (GM-CSF) (rmGM-CSF) and 5 ng/mL recombinant mouse IL-4 (rmIL-4) (both from PeproTech, USA) in 24-well cell culture plates at 37°C and 5% CO_2_. On day 2, 1 mL fresh medium containing rmGM-CSF and rmIL-4 was added. On day 4, all 2 mL of the medium was replaced with fresh medium containing rmGM-CSF and rmIL-4. On day 6, adherent cells were harvested as immature BMDCs and examined under microscopy and flow cytometry for CD11c expression. Then, the BMDCs were stimulated by OVAp and OVAp-CRT in 24-well cell culture plates for 24 h.

### 2.8. Evaluation of DC Antigen-Presenting Ability

The BMDCs were induced as described above and cocultured with T cells isolated from the OT-1 mice by the Pan T Cell Isolation Kit II, mouse (Miltenyi Biotec, Germany). These T cells were labeled with CFSE (carboxyfluorescein succinimidyl ester, Sigma-Aldrich, USA) before being seeded into 96-well cell culture plates. These cells were cocultured at an appropriate DC: T cell ratio of 1 : 10 and then stimulated by OVAp or OVAp-CRT. After 3 days, the CFSE fluorescence intensity in the T cells was measured by Attune NxT flow cytometer (Life Technology, USA).

### 2.9. MTT Assay

Splenocytes isolated from the immunized animals were stimulated with OVA (Sigma-Aldrich, USA) or bovine serum albumin (BSA, Sigma-Aldrich, USA) in a 5% CO_2_ incubator at 37°C. After 3 days, 10 *μ*L 3-(4,5-dimethyl-2-thiazolyl)-2,5-diphenyl-2-H-tetrazolium bromide (MTT, Sigma-Aldrich, USA, 5 mg/mL) was added to the medium, and cells were incubated for 4 h at 37°C. The supernatants were removed, and 100 *μ*L dimethyl sulfoxide was added to each well. The optical density (OD) was measured at 570 nm by Synergy H4 Hybrid Multi-Mode Microplate Reader (BioTek, USA).

### 2.10. Cytotoxicity Assay

Splenocytes isolated from the immunized animals were restimulated in 48-well cell culture plates for 24 h with OVA. Then, these splenocytes were cocultured with CFSE-labeled B16 or B16-OVA cells at a ratio of 25 : 1. After 4 h coculture, the cells were removed to the tube, and propidium iodide (PI, BioLegend, USA) was added. The CFSE and PI double-positive cells were identified by Attune NxT flow cytometer (Life Technology, USA).

### 2.11. Interferon- (IFN-) *γ* ELISpot Assay

The splenocyte IFN-*γ* secretion levels were quantified by using the Mouse IFN-*γ* ELISPOT Set (BD Biosciences, USA) following the manufacturer's protocol. The cells were stimulated by 24 h incubation with OVA peptide 257-264 (SIINFEKL, GenScript, China) before secondary antibody treatment. IFN-*γ* spot-forming cells were counted by an ImmunoSpot Analyzer ELISPOT reader (Cellular Technologies Ltd, USA).

### 2.12. OVA-Specific Antibody Analysis

96-well polystyrene plates were coated overnight at 4°C with OVAp or BSA at 2 *μ*g/mL in carbonate buffer (pH 9.6). The coated plates were incubated with a blocking solution (2% BSA in PBS) for 2 h at 37°C. These wells were washed five times with PBST (PBS containing 0.05% Tween 20), and then, 100 *μ*L diluted mouse serum was added to the wells and incubated for 2 h at 37°C. After five times washes with PBST, the plates were incubated with horseradish peroxidase-labeled goat anti-mouse IgG antibodies (SouthernBiotech, USA) for 1 h at 37°C. The reaction was developed with 100 *μ*L TMB (3,3′,5,5′-tetramethylbenzidine, Sigma-Aldrich, USA) for 5 min and stopped with 100 *μ*L 2 M H_2_SO_4_. The OD was measured at 450 nm by Synergy H4 Hybrid Multi-Mode Microplate Reader (BioTek, USA).

### 2.13. Mouse Immunization

C57BL/6 mice were immunized subcutaneously with PBS alone or with 100 *μ*g OVAp or OVAp-CRT in PBS on day 0 and with 50 *μ*g OVAp or OVAp-CRT on days 7 and 14. On day 21, the serum was collected, and the splenocytes and ILN cells were harvested for analysis.

### 2.14. Tumor Mouse Models

We evaluated the adjuvant efficacy of OVAp-CRT for preventing tumor development. Four groups of mice (*n* = 5) were immunized on days 0, 7, and 14 *via* the hypodermic route. Two groups each were immunized with OVAp-CRT (100 *μ*g/animal) and OVAp (100 *μ*g/animal) on day 0 and with a 50 *μ*g per mouse dose on days 7 and 14. One week after the last immunization, one OVAp-CRT-immunized group and one OVAp-immunized group was challenged with 1 × 10^6^ B16-OVA cells *via* the hypodermic route. The remaining two groups were challenged with 1 × 10^6^ B16 cells as the control. The tumor size and the probability of survival were recorded. The volume was calculated according to the formula (0.5 × length × width^2^). Mice were sacrificed at the ethical experimental endpoint (2 cm^3^ tumor size), and the survival times of mice were recorded and plotted.

### 2.15. BMDC Adoptive Transfer

BMDCs were generated from C57BL/6 mice. On day 6, the BMDCs were harvested and pulsed with 10 *μ*g/mL OVAp or OVAp-CRT at a cell concentration of 2 × 10^6^/mL for 24 h and then washed with PBS three times. C57BL/6 mice were transferred intravenously thrice at a 1-week interval with 1 × 10^6^ protein-pulsed BMDCs per mouse. Next, the mice were challenged with 1 × 10^6^ B16-OVA cells *via* the hypodermic route. The tumor size and the probability of survival were recorded. The volume was calculated according to the formula (0.5 × length × width^2^). Mice were sacrificed at the ethical experimental endpoint (2 cm^3^ tumor size), and the survival times of mice were recorded and plotted.

### 2.16. Statistical Analysis

All statistical analyses were performed with GraphPad Prism 6. Data are presented as mean ± standard error of the mean (SEM). Two groups of independent samples were compared using unpaired Student's *t*-tests. One-way analysis of variance (ANOVA) with Tukey's post hoc test was used to compare multiple groups, and survival curves were obtained using the Kaplan-Meier method and compared by the log-rank test. A *p* value of 0.05 was considered statistically significant.

## 3. Results

### 3.1. CRT/39-272 Promoted DC Maturation, IL-12 Production, and Antigen Presentation *In Vitro*

**Figure 1:**
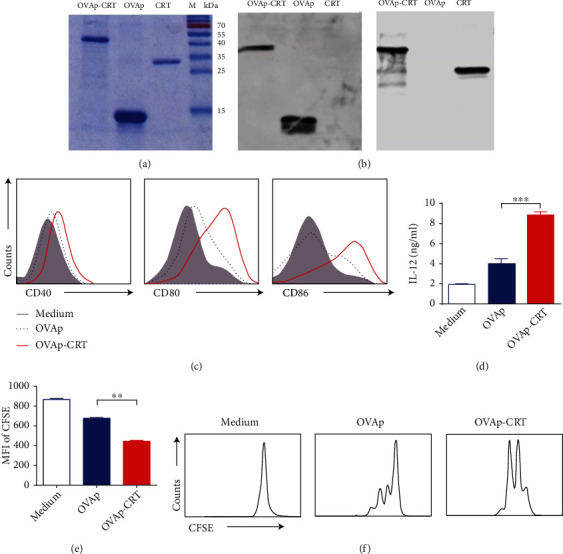
Expression and purification of recombinant proteins and CRT/39-272 promote DC function *in vitro*. (a) Affinity-purified OVAp-CRT (lane 1), OVAp (lane 2), and CRT/39-272 (lane 3) were run in three identical 10% SDS-PAGs. One gel was stained with Coomassie blue. Protein molecular mass markers (M) were loaded in the right lane. (b) Protein bands in the two unstained gels were transferred to PVDF membranes for western blotting with mouse anti-CRT monoclonal antibodies (left) and rabbit anti-OVA polyclonal antibodies (right). (c) Immature BMDCs were stimulated with OVAp or OVAp-CRT for 24 h. The generated DCs, DC-OVAp, and DC-OVAp-CRT were stained with PE-conjugated anti-CD40, anti-CD80, and anti-CD86. (d) IL-12 levels in the supernatants of cultured DCs, DC-OVAp, and DC-OVAp-CRT were analyzed by ELISA. (e and f) BMDCs were incubated with CFSE-labeled pan T cells from OT-I mice at a 1 : 10 ratio in the presence of medium, OVAp, or OVAp-CRT for 3 days. The mean fluorescence intensity (MFI) of the CFSE was analyzed (e) and fluorescence-activated cell sorting (FACS) histograms are shown (f). The results were replicated three times with a consistent trend. Data are representative of at least three independent experiments. ^∗∗^*p* < 0.01. ^∗∗∗^*p* < 0.001.

We prepared expression vector pET28a-CRT/39-272 encoding for murine CRT/39-272 as described previously [11]. We also constructed recombinant OVAp and a His6-tagged OVAp-CRT fusion protein. The three recombinant proteins were successfully expressed in *E. coli* and purified using Ni columns. Coomassie blue-stained SDS-PAGs demonstrated that the resultant products had >90% homogeneity, and the protein was specifically recognized by mouse anti-CRT monoclonal antibodies and rabbit anti-OVA polyclonal antibodies in the western blotting assays (Figures 1(a) and 1(b)).

It has been demonstrated that CRT/39-272 can promote DC maturation [12]. In the present study, we compared CD40, CD80, and CD86 expression levels in OVAp- or OVAp-CRT-stimulated BMDCs. OVAp stimulation did not significantly alter the BMDC CD40, CD80, and CD86 expression levels. In contrast, OVAp-CRT stimulation significantly increased CD40, CD80, and CD86 expression levels in the BMDCs (Figure 1(c)). Similarly, the supernatant of OVAp-CRT-stimulated BMDCs demonstrated significantly increased IL-12 levels (Figure 1(d)). Overall, these data indicate that CRT/39-272 promotes BMDC maturation and IL-12 production *in vitro*.

T lymphocytes are the main targets of DC promotion. Accordingly, we investigated the T cell stimulatory capacities of OVAp-CRT-stimulated BMDCs. BMDCs were stimulated by OVAp and OVAp-CRT for 24 h and cocultured with pan T cells from OT-I mouse spleen. The OVAp-CRT-stimulated BMDCs induced T cell proliferation significantly more potently than OVAp-stimulated BMDCs (Figures 1(e) and 1(f)).

### 3.2. CRT/39-272 Promoted the Maturation of Splenic Conventional DCs (cDCs)

**Figure 2:**
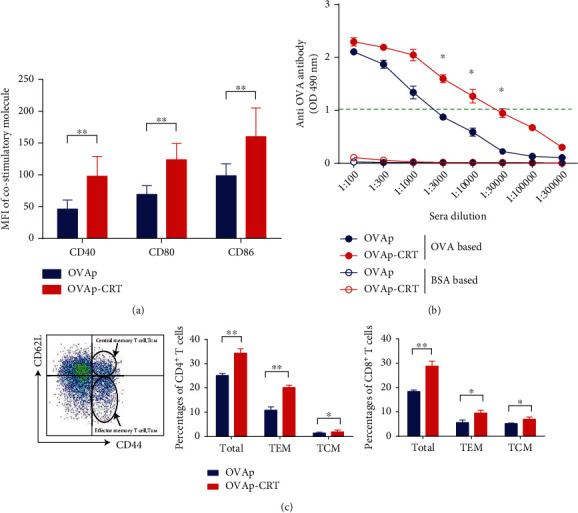
*In vivo* administration of CRT/39-272 induce splenic cDC maturation and enhance OVA-specific T/B cell responses. C57BL/6 mice were treated with OVAp or OVAp-CRT for 21 days. (a) The MFI of CD40, CD80, and CD86 expression on the splenic CD11c^+^ cDCs was analyzed by flow cytometry. (b) Serum OVA-specific IgG concentrations were measured by ELISA. (c) ILN cells were obtained from the immunized mice on day 21, and CD4, CD8, CD44, and CD62L expressions on the ILN cells were analyzed by flow cytometry. The percentage of total CD4^+^ and CD8^+^ T cells and the T_CM_ and T_EM_ cells in CD4^+^ or CD8^+^ T cells. Data are representative of at least three independent experiments. ^∗^*p* < 0.05. ^∗∗^*p* < 0.01.

The previous chapter showed that CRT/39-272 could induce mouse BMDC maturation *in vitro*. Next, we assessed whether CRT/39-272 could also enhance mouse DC maturation *in vivo*. We immunized mice with OVAp or OVAp-CRT and harvested the splenocytes from the immunized mice on day 21. In contrast to OVAp, OVAp-CRT led to a substantial increase in CD40, CD80, and CD86 expressions in splenic CD11c^+^ cDCs (Figure 2(a)). These data indicate that the CRT/39-272 could enhance splenic cDC maturation *in vivo*, and these matured cDCs may enhance antigen-specific T and B cell immune responses.

### 3.3. CRT/39-272 Enhanced OVA-Specific T/B Cell Responses *In Vivo*

Can CRT/39-272-matured cDCs enhance T/B cell immune responses *in vivo*? To answer this question, we examined OVA-specific antibody production and effector T cells in mice immunized with OVAp and OVAp-CRT. On day 21, the mouse serum was analyzed for OVAp-specific IgG. The OVAp-CRT-immunized mice produced remarkably higher amounts of anti-OVAp IgG than the control mice immunized with OVAp alone (Figure 2(b)). We also analyzed the percentage of CD4^+^ and CD8^+^ T cells in the ILN (Figure 2(c)). There was a significant increase in the percentage of total CD4^+^ and CD8^+^ T cells from the ILN of OVAp-CRT-immunized mice. Then, we examined whether CRT/39-272 promoted the generation of central memory (T_CM_, CD44^hi^CD62L^hi^) and effector memory T cells (T_EM_, CD44^hi^CD62L^low^) in OVA-immunized mice based on the surface expression of CD44 and CD62L. As shown in Figure 2(c), CRT/39-272 led to a marked increase in the proportions of CD4^+^ T_EM_/T_CM_ and CD8^+^ T_EM_/T_CM_.

### 3.4. CRT/39-272 Enhanced OVA-Specific T Cell Differentiation and Function *Ex Vivo*

**Figure 3:**
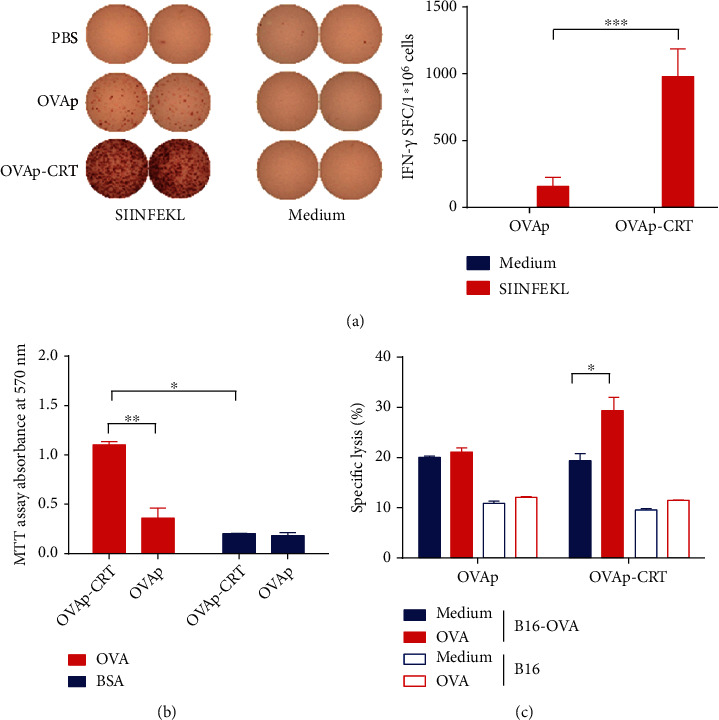
CRT/39-272 exerts an adjuvant effect on OVAp-induced T cell differentiation and function. C57BL/6 mice were immunized subcutaneously with OVAp or OVAp-CRT on days 0, 7, and 14. Splenocytes harvested from immunized mice on day 21. (a) Splenocytes were restimulated with or without SIINFEKL (0.5 *μ*g/mL) for 24 h. IFN-*γ* secreting cells were determined by IFN-*γ* ELISpot assay. (b) Splenocytes were restimulated with OVA or BSA (10 *μ*g/mL) for 3 days. Cell proliferation from restimulated splenocytes was measured. (c) Splenocytes were restimulated with or without OVA (10 *μ*g/mL) for 24 h, and then, CFSE-labeled B16 or B16-OVA cells were added. After 4 h coculture, the cells were removed, and the dead tumor cells were determined by flow cytometry. The results were replicated three times with a consistent trend. Data are representative of at least three independent experiments. ^∗^*p* < 0.05. ^∗∗^*p* < 0.01. ^∗∗∗^*p* < 0.001.

The H2-K^b^-restricted peptide SIINFEKL was used to restimulate splenocytes, and IFN-*γ*-secreting cells were determined by IFN-*γ* ELISpot assay. Splenocytes were harvested and restimulated with SIINFEKL *in vitro* for 24 h and then analyzed for OVA-induced T cell response. As shown in Figures 3(a) and 3(b), splenocytes from the OVAp-CRT-immunized mice demonstrated significantly greater cell proliferation and IFN-*γ* production than those from control mice immunized with OVAp alone. These results indicate that CRT/39-272 can function as an adjuvant by promoting Th-type immune responses. Finally, we tested the cytotoxicity of CTLs against B16 or B16-OVA cells. The splenocytes from immunized mice were restimulated with OVA *in vitro* for 24 h and then cocultured with CFSE-labeled B16 or B16-OVA cells. The percentages of apoptotic B16 or B16-OVA cells were determined by flow cytometry using PI. Coculture with splenocytes from OVAp-CRT-immunized mice, but not splenocytes from OVAp-immunized mice, further increased the percentages of apoptotic B16-OVA cells. There was no significant difference in the percentages of apoptotic B16 cells induced by OVAp- or OVAp-CRT-immunized splenocytes (Figure 3(c)). These data all suggest that CRT/39-272 can enhance antigen-specific T cell immune responses.

### 3.5. OVAp Immunization with CRT/39-272 as an Adjuvant Protected Mice from Challenge by B16-OVA Cells

**Figure 4:**
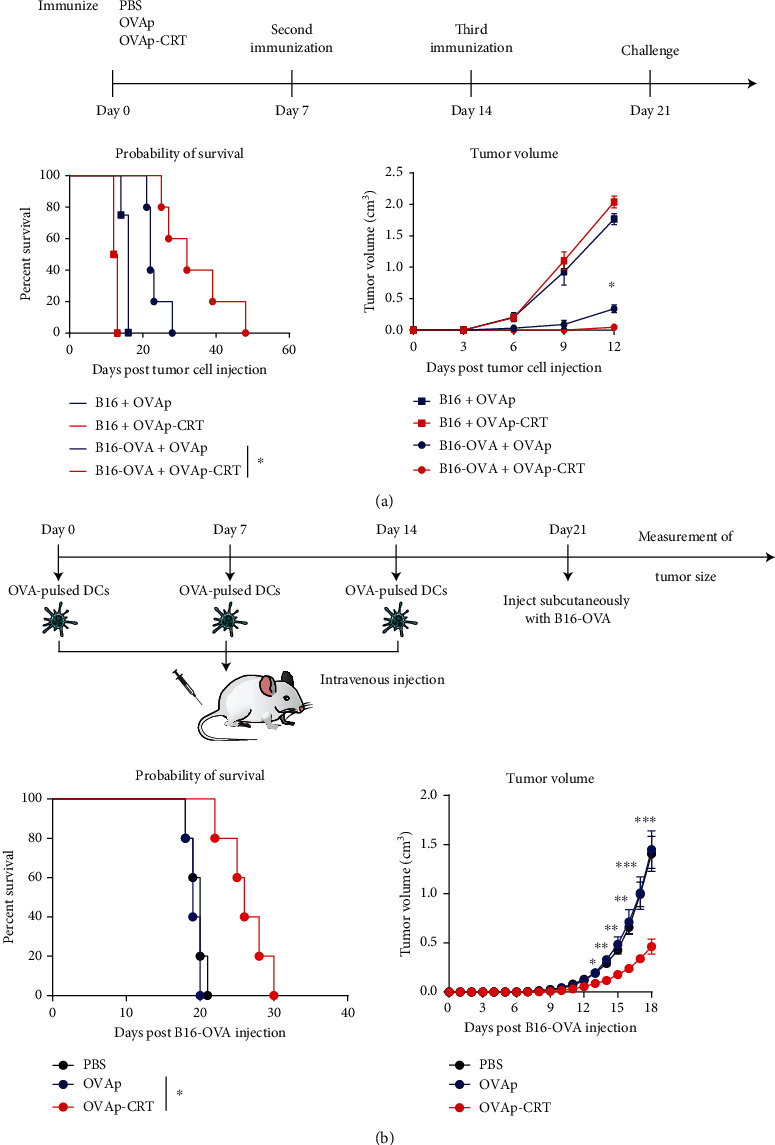
Immunization with OVAp- or OVAp-CRT-pulsed DCs protects mice from challenge with B16-OVA tumor cells. (a) Mice were immunized with OVAp or OVAp-CRT on days 0, 7, and 14. Seven days after the final immunization, the mice were challenged by subcutaneous injection with 1 × 10^6^ control or OVA-transgenic B16 tumor cells (B16 and B16-OVA, respectively). Following B16-OVA or B16 cell inoculation, mouse survival was recorded. Tumor growth was monitored by measuring the tumor diameter every 3 days and recorded as the average tumor diameter. (b) DCs were stimulated with OVAp or OVAp-CRT. Mice were adoptively transferred with OVAp- or OVAp-CRT-pulsed DCs on days 0, 7, and 14. Seven days after the final immunization, the mice were challenged subcutaneously with 1 × 10^6^ B16-OVA tumor cells. Following B16-OVA tumor inoculation, mouse survival was recorded. Tumor growth was monitored by measuring the tumor diameter every 3 days and recorded as the average tumor diameter. *n* = 5 mice per group. Data are representative of at least three independent experiments. ^∗^*p* < 0.05. ^∗∗^*p* < 0.01. ^∗∗∗^*p* < 0.001.

Based on the observation that CRT/39-272 functions as an adjuvant to activate OVA-specific CD4^+^ and CD8^+^ T cells, we investigated whether this response can protect mice grafted with OVA-expressing B16 cells. C57BL/6 mice were immunized subcutaneously with OVAp or OVAp-CRT on days 0, 7, and 14 and were inoculated subcutaneously with B16 or B16-OVA cells on day 21. OVAp-CRT-immunized mice were almost completely protected from the B16-OVA tumor challenge and exhibited smaller tumors and longer survival compared with OVAp-immunized mice. However, the B16-inoculated groups demonstrated no marked tumor growth inhibition or survival improvement (Figure 4(a)).

### 3.6. Adoptive Transfer of DCs Pulsed with OVAp-CRT Protected Mice from B16-OVA Challenge

To assess whether DCs pulsed with OVAp-CRT were capable of inducing an immune response against B16-OVA cells *in vivo*, we immunized C57BL/6 mice with syngeneic DCs that had been pulsed with OVAp-CRT or OVAp. The blank control was PBS-treated DCs. After three rounds of immunization *in vivo*, the mice were inoculated subcutaneously with 1 × 10^6^ B16-OVA cells. The tumor volume was observed daily. As shown in Figure 4(b), mice adoptively transferred with OVAp-CRT-pulsed DCs exhibited smaller tumors and lived longer than mice transferred with OVAp- or PBS-pulsed DCs. The tumor volume and survival of the mice immunized with OVAp- or PBS-pulsed DCs were not significantly different.

## 4. Discussion

DC-based cancer vaccines are based on optimal antigen presentation and T cell activation of DCs. Between 1995 and 2021, many DC vaccines were tested in melanoma, B cell lymphoma, metastatic colorectal cancer, hematologic malignancies, and hepatocellular cancer [27–31]. A critical area in DC vaccine design and translation is the culture condition. As DCs are highly sensitive environmental sensors, the conditions to which they are exposed can strongly impact their function. Another critical area is antigen loading. Many antigen sources have been tested *in vitro*: MHC I-restricted peptides [32], containing peptides [33], autologous tumor cells (lysates and cells), and killed allogeneic tumor cells [34]. Nevertheless, antigens alone might occasionally not mature DCs and enhance antigen-specific T cell responses; many adjuvants were used to promote DC functions. HSPs are known for their adjuvant effect in peptide immunization for initiating specific cellular immune responses against associated antigens. Yanfeng et al. reported that Hsp70, a member of HSPs, could be an adjuvant for enhancing the induction of the carcinoembryonic antigen-specific CD8^+^ CTL response by a DC vaccine [35]. As a tumor HSP, CRT may be an adjuvant of DC vaccines.

CRT is related to tumors. Cell surface CRT (ecto-CRT) can be found in various tumor cell membranes (e.g., melanoma), and sCRT is present in the body fluids of patients with different cancers [8–10]. On apoptotic tumor cells, ecto-CRT activates phagocytic cells to engulf tumor cells [36]. As a molecular chaperone, CRT can also enhance antigen presentation, and ecto-CRT in tumor cells may carry tumor-derived antigens (or antigenic peptides) when it is expressed, which means that CRT can elicit effective tumor antigen-specific adaptive cellular responses *in vivo*. Earlier studies have demonstrated that the CRT N-terminal domain inhibits endothelial cell proliferation and angiogenesis [37]. More recent work has demonstrated that sCRT increases T cell infiltration in tumor tissues by augmenting adhesion molecule expression on endothelial cells [38]. However, CRT function in tumor development *in vivo* remains controversial. Our research has indicated that sCRT may promote B16 melanoma malignancy through myeloid-derived suppressor cells [39].

The physical link between OVAp and CRT/39-272 is necessary for improving immunogenicity, as our previous results have indicated that a mixture of CRT/39-272 and antigen or hapten (e.g., *β*-glucan and peptide of the SARS-CoV spike protein) [13, 40] was no more immunogenic than antigen or hapten alone. Another advantage of OVAp-CRT over OVAp as an immunogen is its better hydrophilicity, where OVAp is less soluble in preparations and is mainly expressed in the inclusion bodies of pET28a-OVAp-harboring bacteria. Therefore, renaturation steps were necessary after Ni column purification, and the resultant product required maintenance at a relatively low concentration to avoid protein aggregation and precipitation. By contrast, OVAp-CRT is a highly soluble polypeptide and is present in the lysate of *E. coli* cells harboring pET28a-OVAp-CRT. The final product is also less likely to form aggregates in PBS.

CRT is constitutively expressed in mammalian cells and can be a protein chaperone in ER. Previously, we demonstrated that CRT/39-272 can activate B cells and macrophages *in vitro* [11], and other researchers have reported that CRT induces DC activation and maturation via TLR4 and CD14 and through the PI3K-Akt signaling pathway [12]. As an adjuvant, CRT/39-272 aided anti-*β*-glucan IgG responses in NOD (nonobese diabetic) mice [40] and enhanced IgG responses to the SARS-CoV spike protein [13]. All these data indicate that CRT/39-272 can function as an exceptional molecular adjuvant of viral, bacterial, and fungal components. Cheng et al. reported that intradermal immunization with a DNA vaccine encoding a fusion protein between CRT, or CRT fragments, and E7 tumor antigen was more efficient for eliciting E7-specific CD8^+^ T cells and protecting against E7-expressing tumors in C57BL/6 mice [41]. CRT protein can also act as an adjuvant to promote DC maturation and enhance antigen-specific CTL responses against non-small-cell lung cancer cells *in vitro*. Unlike common adjuvants, such as incomplete Freund's adjuvant or alum, CRT/39-272 can induce effective CTL responses. Other adjuvants such as liposomal adjuvants, CpG, monophosphoryl lipid, and QS-21 can bind to TLRs to activate DCs; nevertheless, these adjuvants appear to have limited effects on DC maturation, antigen-presenting function, and CTL response induction [26]. By contrast, CRT/39-272 can upregulate CD40, CD80, and CD86 expressions in DCs *in vitro* and *in vivo*. Furthermore, activated DCs can secrete various cytokines, such as IL-12, IL-6, and tumor necrosis factor- (TNF-) *α*, but they also can secrete IL-10, which inhibits T cell immunity [42]. Liu and colleagues reported that CRT/39-272-stimulated DCs demonstrated increased IL-12 secretion and decreased IL-10 secretion.

## 5. Conclusions

We have demonstrated that CRT/39-272 can be an adjuvant *in vivo* to promote DC maturation and enhance antigen-specific T lymphocyte responses, including antigen-specific T cell proliferation, IFN-*γ* secretion, and CTL responses. In mice, OVAp-CRT or OVAp-CRT-pulsed DCs inhibited B16-OVA cell growth and prolonged survival. These results all indicate the excellent adjuvant effects of CRT/39-272.

## Data Availability

The data that supported the findings of this study are available on reasonable request from the corresponding author.
